# Preservation of the middle meningeal artery during unruptured aneurysm surgery: an independent risk factor for postoperative chronic subdural hematoma

**DOI:** 10.3389/fneur.2024.1400788

**Published:** 2024-05-06

**Authors:** Myungsoo Kim

**Affiliations:** Department of Neurosurgery, School of Medicine, Kyungpook National University, Daegu, Republic of Korea

**Keywords:** chronic subdural hematoma (CSDH), unruptured aneurysm, middle meningeal artery (MMA), microsurgical clipping, pterional approach, lateral supraorbital approach

## Abstract

**Background:**

Although microsurgical clipping for unruptured aneurysms has become safer and more efficient with modern neurosurgical advances, postoperative chronic subdural hematoma (CSDH) persists as an underrecognized complication. This study investigated the association between preservation of the anterior branch of the middle meningeal artery (MMA) during surgery and CSDH development.

**Methods:**

We retrospectively reviewed 120 patients who underwent clipping for unruptured aneurysms at Kyungpook National University Chilgok Hospital between May 2020 and July 2023. We evaluated the patients on the basis of surgical approach—lateral supraorbital (LSO) or standard pterional craniotomy—and the status of the MMA postoperatively. We employed pre-and post-operative MR angiography to assess MMA preservation and used follow-up computed tomography scans to monitor CSDH development.

**Results:**

Of the 120 patients, 22 (18.3%) developed CSDH. Univariate analysis revealed that male sex, advanced age, and MMA preservation are risk factors for postoperative CSDH. Multivariate analysis supported these findings, indicating a significant association with the development of CSDH. MMA preservation was reported in 65 patients, of whom 60 and 5 underwent LSO and pterional craniotomy, respectively.

**Conclusion:**

Preservation of the anterior branch of the MMA during unruptured aneurysm surgery is a risk factor for postoperative CSDH development. Advanced age and male sex also contribute to the increased risk. These findings highlight the need for further investigation into surgical techniques that could mitigate postoperative CSDH development.

## Introduction

Modern advancements in neurosurgical technology have significantly enhanced the safety and efficacy of microsurgical procedures for unruptured aneurysms. As minimally invasive aneurysm surgery becomes more common, recent data indicate a major surgical morbidity of less than 1% for unruptured aneurysms measuring below 10 mm (1–3). However, postoperative subdural hygromas transitioning to chronic subdural hematomas (CSDH) remain an underestimated complication. A recent study showed that male sex and advanced age are risk factors for CSDH formation following unruptured aneurysm surgery (4, 5).

The middle meningeal artery (MMA), which is commonly cauterized during pterional craniotomy, supplies the outer membrane of CSDH and significantly contributes to the neovascularization of the membrane associated with CSDH (6, 7). Furthermore, previous studies have shown that CSDH mainly occurs in the anterior region and is predominantly supplied by the anterior branch of the MMA, and MMA occlusion during burr hole procedures can reduce the recurrence rates of CSDH (6).

On the basis of these observations, we postulate that MMA preservation during unruptured aneurysm surgery may correlate with CSDH formation. This study aimed to evaluate the risk factors for CSDH in patients undergoing microsurgical clipping for unruptured aneurysms, focusing on the impact of MMA preservation.

## Materials and methods

This study was reviewed and approved by the Ethics Committee of Kyungpook National University Chilgok Hospital (IRB No. 2023-10-027).

### Patient population

This study enrolled 120 patients who underwent surgical treatment for unruptured aneurysms at Kyungpook National University Chilgok Hospital from May 2020 to July 2023.

The inclusion criteria were as follows: (1) age >18 years; (2) diagnosis of unruptured, nongiant aneurysms arising at the anterior circulation of the circle of Willis, including the supraclinoid internal carotid artery (ICA), anterior cerebral artery (ACA), and middle cerebral artery (MCA); (3) treatment of unruptured aneurysms via lateral supraorbital (LSO) or standard pterional craniotomy; (4) preoperative magnetic resonance angiography (MRA).

The exclusion criteria were (1) diagnosis of ruptured aneurysm with subarachnoid hemorrhage, (2) aneurysm arising at the distal ACA requiring interhemispheric approach, (3) aneurysm arising at the posterior circulation, and (4) no available preoperative MRA data.

### Surgical procedure

**Figure 1:**
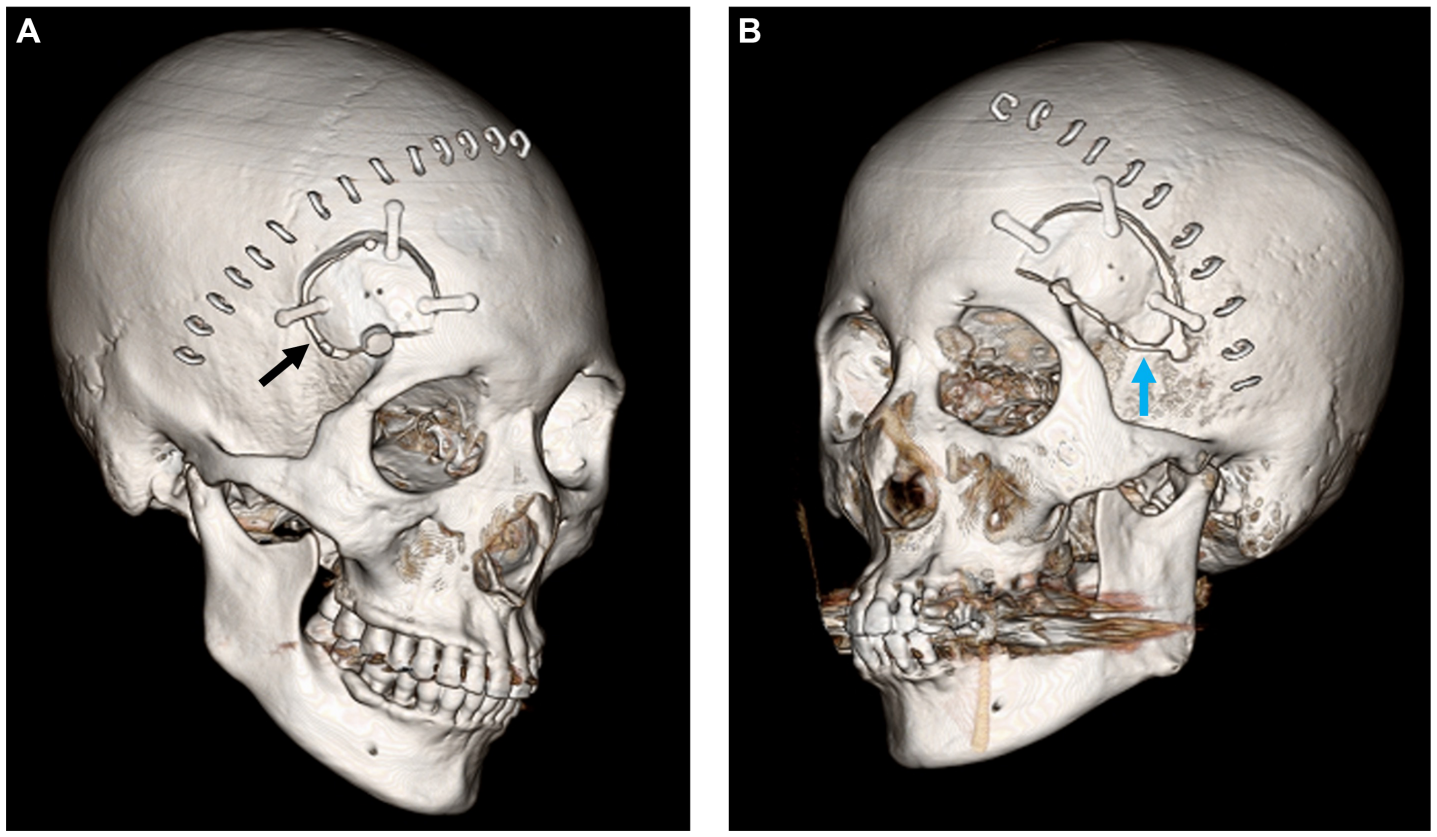
**(A)** Postoperative 3D CT image of lateral supraorbital craniotomy (black arrow). **(B)** Postoperative image of pterional craniotomy with sphenoid bone working (blue arrow).

In this study, either the standard pterional or the LSO approach was employed (3, 8, 9). The pterional approach was performed in a standard manner (Figure 1B), routinely including surgical occlusion of the anterior branch of the MMA during sphenoid ridge bone work. The LSO approach included small craniotomy (diameter of below 4 cm) with MMA preservation, as previously reported by the authors of the present study (Figure 1A) (3, 10). In both surgical techniques, the inner edge of the craniotomy above the orbital rim was refined, and frontal floor protrusions were smoothed for better subfrontal access. After opening the dura, a brain spatula was directed over the base of the frontal lobe to the carotid and optic nerve cisterns. Draining of CSF from these cisterns ensured safe and sufficient frontal lobe retraction.

### Data collection

The medical records and radiological findings of the patients were examined to gather pertinent clinical and imaging data. All patients discontinued antiplatelet medication before surgery.

Routine brain computed tomography (CT) scans were performed on the first day postoperatively, upon discharge (between days 5 and 7), and 3 weeks postoperatively. When patients revealed any neurological symptoms or enduring headaches or any intracranial complications, further follow-up CT scans were performed.

A CSDH was defined as a crescent-shaped isodense or slightly hyperdense extra-axial collection in the frontoparietal lesion, which was transformed from the subdural hygroma, with a maximum thickness of >3 mm. The CSDHs were monitored via CT scans every 1–3 months until their resolution (either spontaneously or after surgical treatment).

To confirm surgical occlusion of the anterior branch of the MMA, preoperative and routine postoperative MR angiography was retrospectively analyzed. In CT angiography, if the running of the MMA is confirmed in the axial view, the distinction may be ambiguous due to interference from bone contrast and plates following craniotomy; therefore, additional routine postoperative MR angiography was employed in this study. In the axial view of MR angiography, the anterior branch of the MMA near the sphenoid ridge was identified to confirm the presence of coagulation compared with the preoperative MR angiography. All angiographic images were reviewed by one neurosurgeon (MK).

### Statistical analysis

Statistical analyses were conducted using IBM SPSS Statistics for Windows (version 25.0; IBM Corp., Armonk, NY, United States). Student’s *t*-test or Mann–Whitney *U* test was employed for continuous variables. The chi-squared test and Fisher’s exact test were employed for categorical variables, which were expressed as numbers and percentages. Univariate and multivariate analyses were sequentially conducted to analyze the risk factors for CSDH formation.

## Results

The clinical characteristics of the 120 patients who underwent craniotomy for an unruptured aneurysm in the anterior circulation are presented in Table 1. Among them, 83 were women and 37 were men, and their mean age was 64.6 ± 8.7 years.

**Table 1:** Clinical characteristics of 120 patients who underwent microsurgical clipping for an unruptured intracranial aneurysm.

Characteristics	Number of patients (%)
Male	37 (30.8)
Mean age, years (SD)	64.6 ± 8.7
Hypertension	69 (57.5)
Diabetes	26 (21.6)
*Location of the aneurysm*	
Group A	22 (18.3)
ICA aneurysm	21
Proximal A1 aneurysm	1
Group B	25 (20.8)
AcoA aneurysm	23
AcoA and ICA aneurysms	2
Group C	54 (45)
MCA aneurysm	54
Group D	15 (12.5)
AcoA and MCA aneurysms	7
ICA and MCA aneurysms	8
*Surgical approach*	
Pterional	60 (50)
Lateral supraorbital	60 (50)
Preservation of the anterior branch of MMA	65 (54.1)
Burr hole procedure	3 (2.5)

The patients were divided into the following groups based on the extent of arachnoid dissection: Group A, patients with ICA aneurysm (*n* = 21) and proximal A1 aneurysm (*n* = 1); Group B, patients with AcoA aneurysm (*n* = 23) and concomitant aneurysm arising at ICA and AcoA (*n* = 2); Group C, patients with MCA aneurysm (*n* = 54); and Group D, patients with concomitant aneurysm arising at AcoA and MCA (*n* = 7) and concomitant aneurysm arising at ICA and MCA (*n* = 8).

### Postoperative CSDH

**Figure 2:**
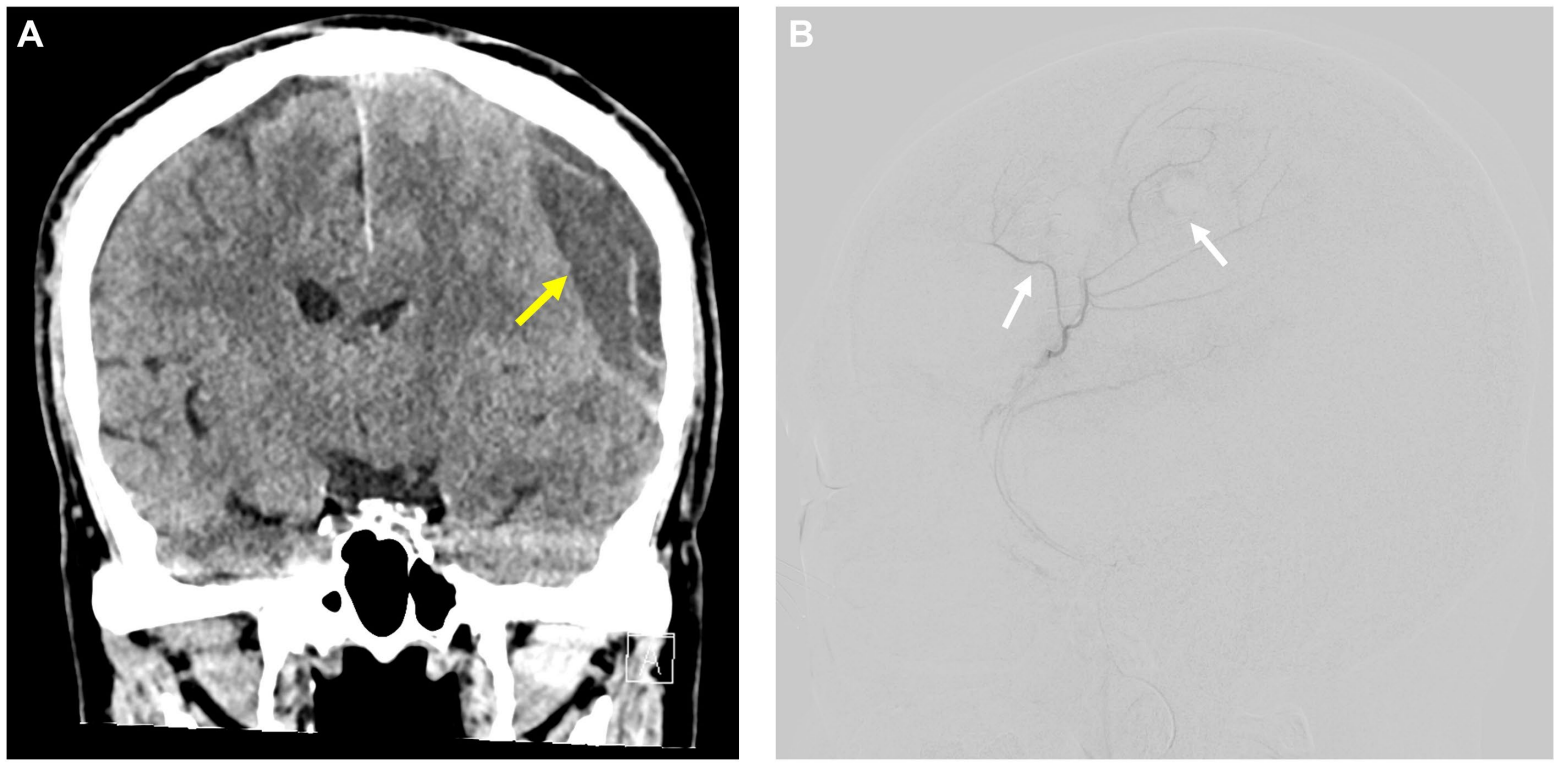
**(A)** Recurrent postoperative CSDH following unruptured aneurysm surgery (yellow arrow). **(B)** Superselective angiography of the anterior branch of the MMA in a patient with recurrent postoperative CSDH showing a widespread capillary network aligned with the microvascular structures found in the external layer of the chronic subdural hematoma (white arrow).

Of the 120 patients, 22 (18.3%) were diagnosed with CSDH. Of these 22 patients, 19 (%) experienced spontaneous resolution of CSDH, as observed in follow-up CT scans. Due to neurological deterioration, three patients underwent surgical burr hole drainage, all of whom had preserved MMA. One patient experienced CSDH recurrence and subsequently underwent reoperation and transfemoral angiography for MMA embolization (Figure 2A). Superselective angiography of the anterior branch of the MMA showed a diffuse vascular network, consistent with microcapillaries, in the outer membrane of the CSDH (Figure 2B). After MMA embolization and repeated burr hole drainage, the patient recovered without any neurological deficit.

### Identification of the middle meningeal artery

**Figure 3:**
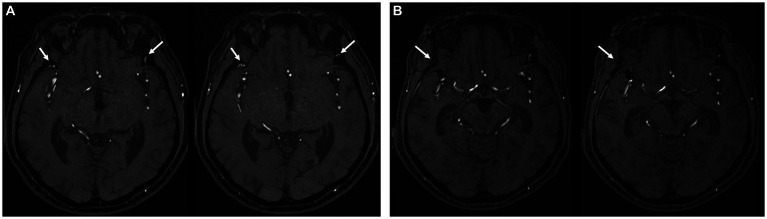
**(A)** Preoperative MR angiography showing the bilateral anterior branch of MMA (white arrow). **(B)** Postoperative MR angiography showing obliteration of the right anterior branch of MMA after pterional craniotomy (white arrow).

This study investigated the preoperative presence of the anterior division of the MMA in all 120 patients in the axial view of MR angiography. The imaging outcomes, showing the pre-and postoperative states of the MMA, are presented in Figures 3A,B. Preservation of the anterior branch of the MMA after unruptured aneurysm surgery was confirmed in 65 patients. Specifically, 60 of these patients underwent LSO craniotomy, all of whom demonstrated postoperative MMA preservation. Furthermore, despite the proximity of the sphenoid ridge bone work to the anterior division of the MMA during pterional craniotomy, five patients still exhibited postoperative preservation of the anterior division of the MMA.

### Risk factors for chronic subdural hematoma

Univariate analysis revealed that male sex (*p* = 0.008), advanced age (*p* < 0.001), LSO surgical approach (*p* = 0.005), and MMA preservation (*p* = 0.004) were all risk factors for postoperative CSDH. However, aneurysm location according to the extent of arachnoid dissection was not a significant risk factor in this study (*p* = 0.329) (Table 2).

**Table 2:** Risk factors for chronic subdural hematoma following unruptured aneurysm surgery on univariate analysis.

Characteristics	Chronic subdural hematoma	Nonchronic subdural hematoma	*p*-value
Patient	22	98	
*Sex*			
Male	12	25	0.008
Age >65	17	35	<0.001
Aneurysm location			0.329
Group A	6	16	
Group B	5	20	
Group C	7	51	
Group D	4	11	
Surgical approach			0.005
Pterional	5	55	
Lateral supraorbital	17	43	
MMA preservation	47	18	0.004

Similarly, multivariate analysis revealed that male sex [odds ratio (OR), 5.535; 95% confidence interval (CI), 1.756–17.451; *p* = 0.003], advanced age (OR, 7.588; 95% CI, 2.262–25.452; *p* = 0.001), and MMA preservation (OR, 4.698; 95% CI, 1.343–16.435; *p* = 0.015) were risk factors for CSDH (Table 3).

**Table 3:** Risk factors for chronic subdural hematoma following unruptured aneurysm surgery on multivariate analysis.

Variable	OR	95% CI	*p*-value
Male	5.535	1.756–17.451	0.003
Age >65	7.588	2.262–25.452	0.001
MMA preservation	4.698	1.343–16.435	0.015

### Clinical outcomes

Among the 22 patients who developed CSDH, 19 experienced spontaneous resolution without significant neurological deficits and recovered without any neurological impairment. Of them, three required surgical intervention for CSDH and recovered well, although one experienced CSDH recurrence.

## Discussion

Postoperative CSDH is an underrecognized complication of unruptured aneurysm clipping surgery. Previous studies have shown that advanced age, male sex, and extensive arachnoid dissection are risk factors for postoperative CSDH (4, 5, 11–13). Similarly, the present study demonstrated that advanced age and male sex are risk factors for CSDH.

Park et al. (4) reported on the incidence of chronic subdural hematoma (CSDH) and subdural hygroma after unruptured cerebral aneurysm surgeries. However, that analysis was imbalanced due to the predominant use of the supraorbital approach in 79.7% of cases, as opposed to the pterional approach in only 20.3%. In addition, the documentation of middle meningeal artery (MMA) preservation was not methodically executed in that study.

This study is unique in that it investigated the implications of preserving the anterior branch of the MMA during unruptured aneurysm clipping and its association with the incidence of postoperative CSDH.

The underlying mechanisms of CSDH formation remain unclear. However, it seems to stem from the detachment of the border cell layer within the dura, initiating a series of healing processes, such as cell proliferation at the dura border, granulation tissue formation, and macrophage activity (7, 14). This ultimately results in neovascularization of the hematoma membrane.

Previous studies have reported that vessels from the MMA can pass through the dura mater and link with emerging neovessels in the external CSDH membrane. Consequently, MMA appears to have a significant influence on the genesis of CSDH and its recurrence, given the vulnerability of these neovessels (6). There is a growing belief that primary expansion and reoccurrence of CSDH are influenced by blood supply to these membranes. This understanding has prompted the use of endovascular embolization techniques targeting MMA as a promising strategy to mitigate recurrence following hematoma drainage (15, 16).

Pterional craniotomy is a commonly used surgical approach that allows access to the anterior cranial fossa and circle of Willis (8, 9, 17, 18). A crucial phase of this operation is the meticulous drilling of the sphenoid ridge, which expands the surgical field. The MMA enters the middle cranial fossa by passing through the foramen spinosum. After entering the middle cranial fossa, the MMA courses anterosuperiorly over the greater wing of the sphenoid bone. It then divides into two main branches: anterior and posterior. The anterior branch courses over the pterion, running forward and medially on the sphenoid ridge (19, 20). By beveling the prominence of the sphenoid ridge, a surgeon can enhance visibility and access to the skull base. During this step, the anterior branch of the MMA is cauterized.

Haldrup et al. (6) reported that CSDH mainly occurs in the anterior region and is predominantly supplied by the anterior branch of the MMA. They also found that deliberate occlusion of the anterior branch of the MMA during a burr hole procedure can reduce the recurrence rates of CSDH, suggesting that similar MMA coagulation during pterional craniotomy influences the occurrence of postoperative CSDH following unruptured aneurysm surgery (6). This finding indicates the potential impact of anterior MMA management on postoperative outcomes.

The present study demonstrates that preservation of the anterior branch of the MMA during unruptured aneurysm surgery is a risk factor for postoperative CSDH.

According to our data, five patients had preserved the anterior branch of the MMA despite routine sphenoid ridge bone work during pterional craniotomy. Due to the retrospective nature of this study, it is unclear how reliably MMA was coagulated during pterional craniotomy. In these five patients, the pterional approach could have been performed without MMA coagulation or the MMA flow could have been restored despite coagulation.

In previous study, Park et al. (4) identified extensive arachnoid dissection as a contributing factor to CSDH development. Furthermore, Han et al. (5) have associated extensive arachnoid dissection with the occurrence of subdural hygroma and found that the volume of collected subdural fluid is a predictor of CSDH. However, in the present study, we did not find a direct association between the degree of arachnoid dissection and the incidence of CSDH.

Efforts to prevent postoperative CSDH from subdural hygroma have yielded promising outcomes through obstruction of the CSF—subdural space interface. This was achieved by applying fibrin glue to close the arachnoid membrane in the Sylvian fissure during aneurysm surgery (12). Nonetheless, there have been reports of allergic reactions to the fibrin adhesive used in arachnoid plasty, and a comprehensive evaluation of the advantages and potential risks based on extensive case studies is still lacking (21).

This study has several limitations. First, the retrospective nature of the data review from a single institution could introduce selection bias, potentially affecting the generalizability of the results. Second, the relatively small sample size limits the statistical power of our findings and may not sufficiently represent the broader patient population. Finally, variations in surgical technique and individual surgeon experience could have influenced the outcomes, although we attempted to mitigate this by having all surgeries performed by experienced neurosurgeons. Despite these limitations, our findings might provide valuable insights into the risk factors associated with CSDH after unruptured aneurysm surgery.

## Conclusion

Preservation of the anterior branch of the MMA during unruptured aneurysm surgery is a risk factor for postoperative CSDH development. Advanced age and male sex also contribute to increased risk. These findings may provide useful information for predicting postoperative CSDH following unruptured aneurysm surgery. Further studies are warranted to elucidate these findings.

## Data availability statement

The original contributions presented in the study are included in the article/supplementary material, further inquiries can be directed to the corresponding author.

## Ethics statement

The studies involving humans were approved by the Ethics Committee of Kyungpook National University Chilgok Hospital (IRB No. 2023-10-027). The studies were conducted in accordance with the local legislation and institutional requirements. Written informed consent for participation was not required from the participants or the participants’ legal guardians/next of kin because of the retrospective nature of the study.

## Author contributions

MK: Conceptualization, Data curation, Formal analysis, Writing – original draft, Writing – review & editing.
